# Thermal Symmetry Between Residual and Intact Limbs in Individuals with Lower Limb Amputation: Resting and Post-Activity Conditions

**DOI:** 10.3390/healthcare14070861

**Published:** 2026-03-27

**Authors:** Senay Cerezci-Duygu, Sevilay Seda Bas, Bahar Anaforoglu

**Affiliations:** 1 Department of Orthotics and Prosthetics, Gülhane Faculty of Health Sciences, University of Health Sciences, Ankara 06010, Türkiye; 2 Department of Physiotherapy and Rehabilitation, Faculty of Health Sciences, Ankara Yıldırım Beyazıt University, Ankara 06760, Türkiye; ssbas@aybu.edu.tr (S.S.B.); baharkulunkoglu@aybu.edu.tr (B.A.)

**Keywords:** amputation, residual limb, temperature, skin surface

## Abstract

**Highlights:**

**What are the main findings?**

- Regional skin temperature patterns differ between residual and intact limbs, indicating asymmetrical physiological adaptation following transtibial amputation.
- The residual limb demonstrates a limited thermal response to functional activity in contrast to the intact limb, suggesting altered vascular or muscular responses.

**What are the implications of the main findings?**

- Region-specific skin temperature assessment may provide a practical, non-invasive tool for monitoring rehabilitation and identifying altered physiological responses after amputation.
- Incorporating region-specific thermal assessment into routine rehabilitation may support individualized prosthetic and exercise interventions.

**Abstract:**

**Background and Objectives**: Individuals with lower limb amputation are at increased risk of developing post-amputation complications that may affect rehabilitation outcomes. Assessing thermal symmetry between the residual and intact limbs, as well as activity-related temperature changes, represents a non-invasive and cost-effective approach for preliminary clinical evaluation. This study aimed to investigate regional skin temperature symmetry between residual and intact limbs at rest and following activity, and to examine whether the presence of phantom limb sensation or pain influences thermal patterns. **Methods**: Twenty-three individuals with unilateral lower limb amputation (mean age: 42.2 ± 15.1 years) participated in this cross-sectional study. The presence of phantom limb sensation and pain was recorded. Skin temperature measurements were obtained using a non-contact infrared thermometer under two conditions: “resting” and “post-activity” following a 10 min self-selected walking task, conducted after prosthetic removal. Measurements were acquired from the patella, tibialis anterior, and distal points of both the residual and intact limbs in both conditions. **Results**: Significant inter-limb differences were observed in the patellar and tibialis anterior regions, with higher temperatures at the patella and lower temperatures at the tibialis anterior on the residual limb. No significant differences were detected at the distal regions under either condition. Post-activity temperature increases were observed in the tibialis anterior and distal regions of the intact limb, whereas no comparable adaptation was observed on the residual limb. Thermal profiles did not differ between participants with and without phantom limb pain or sensation. **Conclusions**: These findings demonstrate that skin temperature dynamics in individuals with amputation are region-specific and influenced by functional activity. The absence of post-activity thermal adaptation in the residual limb may have clinical implications for rehabilitation monitoring. Incorporating regional thermal assessment into routine evaluation may support individualized rehabilitation strategies following lower limb amputation.

## 1. Introduction

Following lower limb amputation, differences are observed in both the intact and residual limbs to compensate for the loss of sensorimotor function [1,2,3]. Soft tissue changes in skeletal muscles have been reported to occur rapidly after amputation. In the residual limb, the cross-sectional area of muscles, muscle-fat percentage, and strength of both the damaged and intact muscles are reduced compared to the intact limb [3,4,5]. Additionally, the quadriceps muscle on the intact side has been found to be atrophied compared to healthy reference groups. Generally, the load-bearing capacity of the residual limb decreases after amputation, resulting in a proportionally greater load transferred to the intact limb during walking [2,6,7].

Various degenerative disorders are frequently observed in both intact and residual limbs due to the effects of defined musculoskeletal changes and altered movement patterns. It was stated that degenerative disorders are associated with physiological factors such as knee vascularization [8]. Although there is evidence suggesting a potential causal relationship between vascular pathology and knee osteoarthritis, a small number of researchers using advanced laboratory methods have proposed that ischemia may trigger anterior knee pain. The decrease in knee skin temperature (Tsk) observed in these patients with ischemia is thought to be caused by vascular impairment [9]. Along with this finding, laboratory and clinical tests have reported a correlation between cooler knees and cases of arterial occlusion in the popliteal fossa [9,10,11]. These findings suggest that vascular pathologies may alter the Tsk and pain profiles.

Previous studies have indicated that infrared thermal imaging has the potential to detect physiological changes that cause changes in Tsk [12,13]. Infrared thermal imaging was reported to be slightly more responsive than other techniques and has the advantage of being quicker to operate and more portable [14]. The method of assessing Tsk in the knee region using an infrared handheld digital thermometer has been validated through comparison with a thermal imaging camera [15]. Typically, Tsk patterns in healthy lower limbs are symmetric. However, a notable Tsk disparity between the two limbs is a reliable indicator of underlying pathology [16]. One study reported that the distal residual limb in individuals with amputation is colder than the corresponding region of the contralateral intact limb [17]. To the best of our knowledge, no studies have comprehensively evaluated and compared the Tsk profiles of the lower limbs, especially the knee joint, in amputee patients. In recent years, skin temperature asymmetry has also been investigated in individuals with unilateral neurological disorders such as stroke and brain injury [18,19]. These studies demonstrated that altered autonomic regulation, impaired muscle activation, and vascular adaptations contribute to measurable inter-limb thermal differences. Such findings suggest that temperature asymmetry reflects broader neuromuscular and metabolic adaptations rather than purely local tissue effects. Therefore, examining thermal symmetry in individuals with limb loss may provide comparable insight into peripheral physiological adaptation following disruption of normal sensorimotor function.

In addition to its potential to indicate various vascular and musculoskeletal system pathologies, researchers have raised the issue of the relationship between thermal anomalies in the residual limb and phantom limb sensations [17,20]. Previous studies have presented preliminary data suggesting that changes in pain intensity may correspond to changes in surface blood flow, as measured by thermal assessment, and that the intensity of a specific phantom pain feature (“burning pain”) may inversely correlate with temperature in the residual limb compared to the unaffected limb [21]. Other studies have suggested that amputees with phantom pain exhibit differences in limb temperature compared to those without and that the corresponding region of the healthy limb is relatively cooler [20]. Investigating thermal changes in the lower extremities following amputation may contribute to a better understanding of vasomotor abnormalities and the pathophysiology of relevant clinical features, such as phantom pain.

Based on the existing evidence, evaluating thermal symmetry between the residual and intact limbs, as well as activity-related temperature changes, may provide a simple and non-invasive method for identifying physiological alterations following amputation. We hypothesized that: (1) the residual limb would demonstrate altered skin temperature distribution compared to the intact limb, and (2) temperature adaptation following activity would be attenuated in the residual limb due to changes in vascular and muscular function. Additionally, we explored whether phantom limb sensation or pain influences thermal symmetry patterns.

## 2. Materials and Methods

### 2.1. Protocol and Registration

The study was planned as a cross-sectional study. This study was approved by the Gülhane Scientific Research Ethics Committee of the University of Health Sciences (2025-156). The study was conducted between June and August 2025. The trial was prospectively registered on the National Library of Medicine platform (NCT06258343). The results are reported according to the Consolidated Standards of Reporting Trials statement.

### 2.2. Participants

Inclusion criteria for participation were 18–65 years of age with a unilateral transtibial amputation, at least 1-year postamputation, use of an extremity prosthesis (prosthesis with elevated vacuum or suction suspension, total surface bearing socket, and carbon foot), and ability to walk without assistance. Individuals who had prior orthopedic surgery involving the lower extremities (excluding amputation) were excluded from the study. The demographic and clinical characteristics of the participants are presented in Table 1.

### 2.3. Outcome Measurements

Within the scope of the study, individuals’ descriptive characteristics, information regarding amputation, and prosthetic extremity were recorded. The prevalence of phantom limb sensation and phantom limb pain was investigated. To determine the presence of phantom sensation, patients were asked if they felt any sensation in the amputated part of their limb. To determine the presence of phantom pain, patients were asked if they felt any pain (or an unpleasant sensation) in the amputated part of their limb. The presence or absence of phantom limb sensation and pain was recorded. This was followed by an evaluation of Tsk measurements at rest and after walking (post-activity).

Skin temperature measurements were obtained using a handheld non-contact infrared thermometer (Medisana 48620, Medisana, FTN, Hilden, Germany), which provides point-based skin temperature readings with an accuracy of ±0.3 °C within the clinical measurement range. The Medisana 48620 infrared thermometers comply with the accuracy requirements of the EC-directive 93/42/EEC. The displayed range is 22–42.9 °C for body temperature and 0–100 °C for surface temperature [22]. Morán-Navarro et al. previously demonstrated the device’s validity and reliability for measuring human skin temperature (Tsk) [23].

Measurement procedures were aligned, where applicable, with recommendations from the Thermographic Imaging in Sports and Exercise Medicine (TISEM) consensus to standardize environmental conditions and participant preparation [24]. Other items on the TISEM checklist, such as recommendations to participants regarding the effects of exercise, smoking, alcohol, lotions, and clinical environment factors on Tsk, were not standardized. Nevertheless, participants were informed about these factors prior to the study and asked to consider and/or avoid them as much as possible. To ensure that Tsk was not affected by clothing or prosthetic components, participants rested for 15 min after removing their shorts and silicone liner, prior to data collection. Participants rested in a supine position with their legs extended to a neutral anatomical position, allowing their skin temperatures to stabilize. This resting stabilization period was implemented to minimize the potential influence of prior physical activity, prosthetic compression, and clothing on skin temperature measurements. Allowing a short acclimatization period after prosthesis removal is recommended, as thermal analysis protocols to allow peripheral skin temperature to equilibrate with the ambient environment. In addition, three repeated measurements were obtained at each anatomical point and averaged to reduce random measurement variability and improve the reliability of the recorded temperature values. It was ensured that no physical intervention had been applied prior to this. The ambient room temperature was recorded simultaneously with the Tsk measurements. Measurements were performed in the same indoor clinical environment during each testing session to minimize environmental variability. Although humidity and airflow were not instrumentally controlled, the assessment room conditions remained stable throughout the measurement sessions.

Skin temperature measurements were conducted in a “resting” condition without a silicone liner and in a “post-activity” condition immediately after removing the prosthetic components following a 10 min walk at self-selected walking speed. Post-activity measurements were obtained immediately after prosthesis removal (within approximately 1 min) to capture early thermoregulatory responses. The 10 min walk was designed to represent a standardized functional activity rather than a controlled exercise test; walking speed was self-selected to reflect habitual gait. Measurements were acquired from the patella, tibialis anterior, and distal points of both the residual and intact extremities under resting and activity conditions. The midpoint of the patella was recorded as “patella”; the point 10 cm distal to the tibial tubercle and 2 cm lateral to the anterior border of the tibia, corresponding to the tibialis anterior muscle belly, was recorded as “tibialis anterior”; and measurements taken from the distal end of the stump and the plantar surface of the intact extremity were recorded as “distal”. Measurements were obtained perpendicular to the skin to minimize emissivity error, in accordance with manufacturer recommendations for clinical infrared thermometry devices. The 3–5 cm distance corresponds to the optimal measurement range specified for the device to ensure accuracy. To determine Tsk, three measurements were taken at each point, and the average was recorded as °C. Participants attended a single assessment visit, during which all skin temperature measurements were performed by the same experienced physiotherapist using predefined anatomical landmarks, a fixed measurement distance, and a perpendicular orientation to the skin surface to minimize operator-dependent variability.

### 2.4. Statistical Analysis

Sample size calculations were performed using G*Power software, Version 3.1.9.4 (Heinrich Heine University, Düsseldorf, Germany). The distal stump and contralateral extremity Tsk values (°C) assessed with an infrared thermometer in the study by Harden et al. were used as a reference, and the effect size was estimated to be 0.71 [17]. The required sample size was determined to be at least 21 individuals for a two-tailed hypothesis test and a dependent-groups *t*-test, with >85% power and a significance level of 0.05.

IBM SPSS Statistics, Version 23.0 (IBM Corp., Armonk, NY, USA) was used for statistical analyses of the data. The normality of the dataset was confirmed using analytical methods (Skewness-Kurtosis) [25]. Parametric tests were applied when a normal distribution was detected, whereas non-parametric tests were used when distributional assumptions were not satisfied. Continuous qualitative data were expressed as frequencies and percentages; quantitative data were expressed as means, medians, standard deviations (SD), and interquartile ranges (IQR) for both sets of scores. Tsk of the residual and intact extremities were compared using the dependent samples *t*-test under parametric conditions and the Wilcoxon Signed Ranks Test under non-parametric conditions. In addition, an exploratory subgroup comparison between participants with vascular and traumatic etiologies was conducted to descriptively examine potential differences; these analyses were interpreted cautiously due to the limited sample size. In all analyses, the type 1 error level was accepted as 5%.

## 3. Results

**Figure 1:**
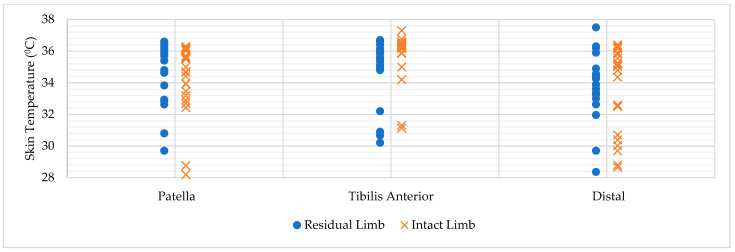
Distribution of Individual Skin Temperature Values Under Resting Conditions.

A total of 23 participants were included in the analysis (Table 1). The ambient temperature was 27.2 ± 1.9 °C during Tsk measurements. Thermal evaluation outcomes for the residual and intact limbs under rest and post-activity conditions are presented in Table 2. The distribution of individual skin temperature values measured at the patella, tibialis anterior, and distal regions of the residual and intact limbs under resting conditions is illustrated in Figure 1.

**Figure 2:**
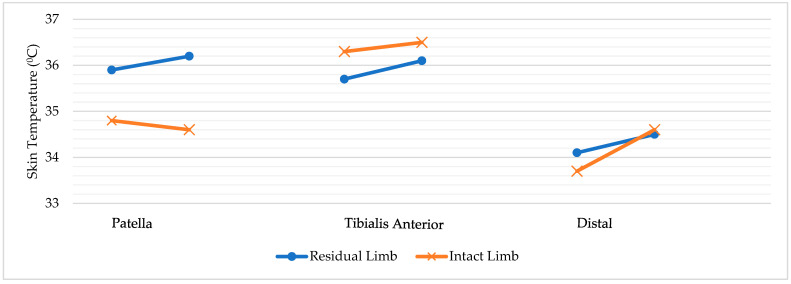
Regional Temperature Changes Between Resting and Post-Activity Conditions.

Pairwise comparisons of residual and intact limbs are presented in Table 3; Pairwise comparisons of rest and post-activity conditions are presented in Table 4. The observed variability is reflected in the dispersion measures (SD and IQR) reported in Table 2, Table 3 and Table 4. Inspection of individual temperature responses indicated variability between participants; however, no consistent subgroup pattern was observed. The individual regional temperature changes between resting and post-activity conditions for residual and intact limbs are illustrated in Figure 2.

Regarding phantom limb sensation and pain, phantom limb sensation was reported in 11 participants (47.8%) and was not reported in 12 participants (52.2%), while phantom limb pain was reported in 9 participants (39.1%) and was not reported in 14 participants (60.9%).

No difference was found between the two groups, with and without phantom limb sensation (n = 11 and n = 12, respectively), in terms of thermal values when comparing the patella, tibialis anterior, and distal regions of the residual and intact limbs (*p* > 0.05). Examining the difference in thermal values between the residual and intact limbs (thermal symmetry) of the two groups, with and without phantom limb sensation, revealed no difference between the two groups (*p* > 0.05).

No difference was found between the two groups, with and without phantom limb pain (n = 9 and n = 14, respectively), in terms of thermal values when comparing the patella, tibialis anterior, and distal regions of the residual and intact limbs (*p* > 0.05). Examining the difference in thermal values between the residual and intact limbs (thermal symmetry) of the two groups, with and without phantom limb pain, revealed no difference between the two groups (*p* > 0.05).

An exploratory comparison of vascular and traumatic amputation etiologies did not reveal statistically significant differences in regional skin temperature (*p* > 0.05). Given the limited number of participants in each subgroup, these findings should be interpreted as descriptive rather than conclusive.

## 4. Discussion

The current study investigated regional thermal symmetry between extremities and peripheral thermal adaptations with activity, as well as the relationship between phantom sensation and/or pain and peripheral thermal profiles in individuals with transtibial amputations. The concept of body temperature symmetry is rarely questioned and is widely regarded as a key indicator of good health. Normative data indicate that bilateral skin temperature differences are usually limited to less than 0.5 °C in healthy subjects, whereas larger asymmetries are considered suggestive of pathological or dysfunctional processes [26,27]. To our knowledge, very little has been discussed about interlimb symmetry in individuals with amputations; however, in recent years, thermal patterns and thermoregulation strategies have been examined by measuring Tsk at the residual limb using different reference points, methods, and research designs [28,29,30,31]. The current study did not classify individuals based on asymmetry thresholds, as the objective was to examine physiological trends rather than diagnose abnormality. Regarding our predefined hypotheses, the findings partially confirmed the first hypothesis, as significant regional differences were observed at the patellar and tibialis anterior levels, but no differences were detected at the distal region. The second hypothesis was supported, as activity-induced temperature adaptation was evident in the intact limb but attenuated in the residual limb. However, the exploratory analysis examining the influence of phantom limb sensation and pain on thermal symmetry was not supported, as no significant differences were observed between groups. The subsequent discussion was conducted within the conceptual framework defined by these headings.

### 4.1. Regional Thermal Asymmetries: Patella and Tibialis Anterior

The current study revealed a considerable regional thermal asymmetry, with higher temperatures detected at the patellar region and lower temperatures at the tibialis anterior region of the residual limb compared to the intact limb. This finding is consistent with the literature describing regional temperature differences within the residual limb. Klute et al. reported significant changes in skin temperatures at different points of the stump in their evaluation within the socket—some areas were warmer due to perfusion patterns and muscle bulk differences [32]. Similarly, Ghoseiri et al. defined unequal distribution of temperature over the transtibial residual limb and reported that certain regions (significantly higher and lower temperatures at its anterior column and distal row, respectively) can differ by over 2 °C from the average residual limb temperature [30]. Theories have been advanced to explain the regional thermal asymmetries between the limbs. These theories include autonomic dysregulation disorders [17] and disembodiment of the missing limb [33]; however, the underlying mechanisms remain unclear and are a subject of debate. To interpret the findings of the current study in the context of existing evidence, the relatively higher temperature observed at the patellar region cannot be attributed to local muscle mass, as this area contains minimal musculature. Rather, this difference may be related to variations in periarticular vascular supply, subcutaneous tissue characteristics, regional load transfer, and potential heat retention effects associated with the socket interface.

### 4.2. Distal Thermal Uniformity

The current study revealed no significant thermal difference between the distal points of both limbs under resting and post-activity conditions. In a study investigating temperature patterns at the distal point of the residual limb in individuals with unilateral transtibial amputations, Harden et al. reported that the distal stump was colder than the corresponding region of the contralateral intact limb [17]. Contrary to the current study’s findings, no significant thermal difference was observed between the distal ends of both limbs during rest and post-activity periods. The discrepancy in thermal value findings between the distal extremities may be due to the different methodologies employed in these two studies. While Harden et al. compared the distal stump to the corresponding area of the intact extremity, the current study compared the temperature at the plantar surface of the intact extremity. Previous studies have emphasized proximal-to-distal gradients in the residual limb, with temperatures typically decreasing distally [32]. Therefore, the absence of distal inter-limb discrepancy in the current study may highlight symmetrical adaptation at the distal extremities.

### 4.3. Thermal Symmetry with Phantom Limb Sensation and Pain

The utilization of temperature assessment in musculoskeletal pathologies has been the subject of prior studies, demonstrating that thermal evaluation may contribute to understanding pain-related physiological changes [34]. Janssen et al. determined that Tsk at the center of the patella differed between patients with and without patellofemoral pain [35], and Wu et al. demonstrated a correlation between a decrease in Tsk and an improvement in pain scores in the case of coccygodynia [36]. Friedrich et al. further suggested that regional temperature assessment may help identify the location of pathology in cases of persistent knee pain, thereby supporting targeted treatment approaches [37]. Along with the studies mentioned, the potential of infrared thermometry for different aspects of pain and its use in targeted interventions has been emphasized. In the current study, observing inter-limb thermal asymmetry at the patellar and tibialis anterior regions was therefore considered relevant for exploring possible alterations in peripheral physiological regulation following amputation. Musculoskeletal pain was not evaluated in the present study; however, the relationship between phantom sensation and pain, as well as the asymmetric pattern, was assessed. In contrast to Katz et al., who discovered that skin temperatures at the stump were significantly lower than those at the contralateral limb in individuals experiencing phantom sensations (painful or non-painful), but not in those without phantom phenomena [38], in the current study, no significant differences in thermal profiles were observed between groups with and without phantom sensations or pain. The absence of association may reflect central mechanisms, such as cortical reorganization and neuroplastic adaptation, that are not necessarily reflected in peripheral thermal changes [39]. This finding suggests that phantom phenomena may be primarily driven by central nervous system processes rather than peripheral physiological alterations detectable through skin temperature assessment. Additionally, methodological variations, sample characteristics, or developing prosthetic technologies may have contributed to this outcome, potentially leading to a more effective restoration of thermoregulatory balance. Katz et al. documented long-term temperature changes linked with sympathetic vasoconstriction; however, activity and modern prosthetic materials may have reduced these differences.

### 4.4. Differential Adaptation During Activity

The current study demonstrated that the activity resulted in substantial increases in temperature in the tibialis anterior and distal regions of the intact limb; however, no significant thermal response was observed at the residual limb with activity. Since walking intensity was not measured instrumentally, it should be noted that physiological load may vary among participants. Furthermore, when evaluating physiological responses, it is known that skin temperature responses to exercise are known to be biphasic. Immediately after activity, temperature may decrease due to vasoconstriction and evaporative cooling, followed by a delayed increase associated with reactive vasodilation and metabolic recovery [40]. The timing of our measurements—taken shortly after prosthesis removal—may reflect this later thermoregulatory phase rather than the initial cooling response described in other protocols. Similarly, Klute et al. investigated the effect of activity on residual limb skin temperatures and found that walking significantly increased skin temperatures within the prosthesis [32]. While this result conflicts with the current study’s findings, we presume that methodological discrepancies, such as measuring temperatures within the prosthesis and using distinct prosthetic suspensions, may have led to this discrepancy. Hasegawa et al. reported significant differences in sweating and skin blood flow between the intact and residual limbs from the 5th to the 60th minute of exercise [41]. As in current study, a marked increase in thermoregulatory indicators, such as sweat gland activity and skin blood flow rate, was observed in the intact limb compared with the residual limb. These findings may provide evidence for the previously proposed role of the intact limb in facilitating compensatory sweating and thermoregulatory function for the cut side in amputees [42]. The intact limb’s temperature rise indicates active thermogenesis via muscular exertion and vasodilation; considering all the evidence, the residual limb’s relative thermal blunting could derive from compromised vascular reactivity, reduced muscle mass, or altered insulating properties of the residual limb-socket interface. Also, this blunted response may represent altered vasomotor regulation or reduced metabolic activation, although this interpretation remains hypothetical without direct hemodynamic measurements. The observed attenuation of temperature response in the residual limb should therefore be interpreted as a potential thermoregulatory pattern rather than definitive evidence of a specific physiological mechanism.

Although several of the observed differences reached statistical significance, their magnitude was relatively small (approximately 1 °C), and this should be interpreted cautiously in terms of clinical relevance. Previous normative investigations have shown that, in healthy individuals, side-to-side skin temperature variation is typically minimal, and deviations exceeding this physiological range may indicate altered tissue function rather than normal thermoregulatory behavior [26,27]. In this context, the differences identified in the present study may reflect subtle changes in peripheral thermoregulation rather than overt pathological conditions. To reduce potential methodological bias, measurement conditions were standardized as far as feasible in accordance with established recommendations for skin temperature assessment in sports and clinical settings, including control of participant preparation and environmental influences [24]. Nevertheless, skin temperature remains sensitive to various intrinsic and extrinsic factors—such as ambient temperature, recent physical activity, nutritional status, and clothing—which cannot be completely controlled in clinical environments. Although ambient temperature was monitored and recorded during all measurements, stricter environmental control under laboratory conditions (e.g., controlled humidity or airflow) could further reduce potential variability in skin temperature measurements. Also, in the present study, baseline temperature measurements were obtained during a single assessment session following a standardized acclimatization period rather than across multiple time points. While this procedure is commonly applied in clinical thermometry protocols, it may not fully capture day-to-day variability in resting skin temperature. Accordingly, the recorded values should be interpreted as standardized clinical measurements reflecting physiological adaptation following amputation rather than as long-term physiological baselines or diagnostic thresholds. Future studies conducted under more tightly controlled laboratory conditions and including reference populations are warranted to establish clinically meaningful cut-off values and further clarify the functional relevance of small temperature differences.

A critical consideration in interpreting the results pertains to the impact of the prosthetic systems used. Among the prosthetic systems most frequently utilized in transtibial amputations are silicone roll-on liners and sealing sleeves in combination with liners. Previous research demonstrated that in individuals with amputations, Tsk increases in the prosthesis (in-liner condition) with the use of silicone liners and/or sealing sleeves [43,44]. However, there is a lack of research addressing the following questions: first, whether vascular adaptation occurs in the knee joint of individuals who utilize liner-equipped prostheses over extended periods; and second, the adaptations that can be observed in liner-free conditions. Due to its elastic nature, a silicone liner or sealing sleeve exerts compression on soft tissue during its application. As Dubuis et al. had previously documented, this compression has been reported to cause intermittent occlusion and ischemia, resulting in hypoxia [45]. In the current study, the application of intermittent ischemia by the prosthetic system is hypothesized to be a contributing factor to the significantly lower temperature of the anterior tibialis region in the residual limb during rest periods. In this regard, there is also a need for studies evaluating the long-term effect of prosthetic materials that apply continuous compression and cause intermittent ischemia, such as liners or knee braces, on cutaneous circulation on the residual side and its possible relationship with pathologies.

From a clinical perspective, thermal assessment may be incorporated as a complementary monitoring tool during rehabilitation, allowing clinicians to observe regional physiological adaptation over time rather than to establish diagnostic thresholds. Recent studies have shown that infrared-based skin temperature evaluation can be used to follow physiological adaptation during rehabilitation [46,47]. Collectively, these applications suggest that serial skin temperature measurements may be incorporated into routine rehabilitation assessments to track adaptation trends, detect atypical regional responses, and assist in individualized rehabilitation planning for individuals with amputation. It should also be recognized that individuals with lower limb amputation represent a highly heterogeneous population in terms of etiology, duration of amputation, prosthetic configuration, and activity levels. These factors may influence thermoregulatory responses and contribute to variability in skin temperature measurements between individuals. Therefore, the findings should be interpreted as indicative of overall thermoregulatory tendencies rather than uniform responses across all individuals.

This study has several limitations. First, vascular pathologies are a primary cause of lower extremity amputations, and the present study found that 35% of participants had been amputated due to vascular diseases. Given that vascular integrity and function are considered determinants of Tsks, the lack of standardization of the cause of amputation in the study sample constitutes a limitation. Although exploratory comparisons were performed, particularly between vascular and traumatic etiologies, the number of participants in each subgroup was insufficient to determine whether underlying vascular pathology independently influences thermal responses. Second, the study did not include a non-amputee control group, which limits the ability to define normative reference values or establish clinically meaningful threshold differences. Third, skin temperature measurements were obtained using point-based infrared thermometry, which may involve a degree of operator dependency. Although all measurements were conducted by a single trained assessor following a standardized protocol with predefined anatomical landmarks and controlled measurement distance, formal intra- or inter-rater reliability analyses were not performed. Future studies should incorporate reliability testing to strengthen methodological rigor. Fourth, phantom limb sensation and pain were assessed using a binary self-report (presence/absence) rather than validated scales evaluating intensity, duration, or qualitative characteristics. This simplified approach may have limited the sensitivity to detect potential associations between pain-related phenomena and thermal patterns. Finally, the cross-sectional design precludes conclusions regarding causality or longitudinal adaptation. Larger prospective studies incorporating control groups, standardized environmental controls, and multimodal physiological assessments are needed to better clarify the mechanisms underlying thermal asymmetry following amputation.

## 5. Conclusions

The current study has demonstrated regional thermal asymmetries between the intact and residual limbs of individuals with transtibial amputation. Specifically, the patellar region of the stump was significantly warmer compared to the intact limb, while the tibialis anterior region was cooler; however, no thermal differences were observed in the distal regions of either limb during rest or post-activity. During activity, expected increases in tibialis anterior and distal temperatures were observed in the intact limb, but the same adaptive response was not seen in the residual limb, which possibly reflected altered vascular and muscle physiology. Importantly, phantom limb sensation and pain were not related to differences in thermal profiles, suggesting that these phenomena do not significantly affect peripheral thermoregulation. This study provides preliminary evidence of region-specific alterations in thermal behavior following transtibial amputation, which should be interpreted considering the methodological limitations and viewed as hypothesis-generating. Future research with larger cohorts, continuous temperature monitoring, and integrated physiological assessments is needed to further clarify the mechanisms underlying thermal asymmetry and adaptation in individuals with amputation.

## Figures and Tables

**Table 1:** Demographic and clinical characteristics of the participants.

Variable		Value (n = 23)
Age (year)		42.2 ± 15.1
BMI (kg/m^2^)		25.3 ± 4.1
Time Since Amputation (year)		12.79 ± 11.2
Sex	Female	3 (13%)
	Male	20 (87%)
Cause of Amputation	Trauma	13 (56.5%)
	Vascular	8 (34.8%)
	Congenital	2 (8.7%)
Functional Level	K3	18 (78.3%)
	K4	5 (21.7%)
Smoking Status	Smokers	4 (17.4%)
	Non-Smokers	19 (82.6%)

Abbreviations: n, number of participants; BMI, body mass index. Values are expressed as mean ± standard deviation for continuous variables and absolute frequency (%) for categorical variables.

**Table 2:** Regional thermal evaluation outcomes of residual and intact limbs in resting and post-activity conditions.

		Residual Limb				Intact Limb			
		Data Range	Mean ± SD	Median [IQR]		Data Range	Mean ± SD	Median [IQR]	
Rest	Patella	29.7–36.6	34.9 ± 2	35.9 [33.8–36.4]	†	28.2–36.3	34.2 ± 2.2	34.8 [33.2–37]	‡
	Tibialis Anterior	30.2–36.7	35 ± 2	35.7 [34.9–36.4]	†	31.1–37.3	35.8 ± 1.6	36.3 [35.9–36.5]	‡
	Distal	28.4–37.5	34.1 ± 2.4	34.3 [33–36.2]	†	28.7–36.4	33.7 ± 2.7	34.9 [30.7–35.9]	†
Post- Activity	Patella	28.4–36.8	35 ± 2.2	36.2 [34.2–36.3]	‡	27.5–36.2	34.1 ± 2.2	34.6 [32.9–35.9]	‡
	Tibialis Anterior	29.5–36.7	35 ± 2.4	36.1 [35.1–36.5]	†	32.2–37.5	36 ± 1.2	36.5 [36.2–36.6]	‡
	Distal	28.2–36.9	34.5 ± 2.5	35.5 [33.7–36.2]	†	29–36.8	34.6 ± 2.3	35.8 [32.8–36.3]	†

All measurements are expressed in degrees Celsius (°C). Abbreviations: SD, Standard Deviation; IQR, Inter-Quartile Range. † Normal distribution; ‡ Not normal distribution.

**Table 3:** Regional Tsk Comparison of Residual and Intact Limb.

		Residual Limb (Mean ± SD/Median [IQR])	Intact Limb (Mean ± SD/Median [IQR])	Test Statistic	95% CI of Difference	*p*
Rest	Patella	35.9 [33.8–36.4]	34.8 [33.2–37]	−2.97 ^z^	**-**	0.003 *
	Tibialis Anterior	35.7 [34.9–36.4]	36.3 [35.9–36.5]	−3.41 ^z^	**-**	0.001 *
	Distal	34.1 ± 2.4	33.7 ± 2.7	0.53 ^t^	[−0.63, 1.06]	0.604
Post- Activity	Patella	36.2 [34.2–36.3]	34.6 [32.9–35.9]	−3.01 ^z^	**-**	0.003 *
	Tibialis Anterior	36.1 [35.1–36.5]	36.5 [36.2–36.6]	−2.83 ^z^	**-**	0.005 *
	Distal	34.5 ± 2.5	34.6 ± 2.3	−0.16 ^t^	[−0.87, 0.16]	0.872

All measurements are expressed in degrees Celsius (°C). Abbreviations: SD, Standard Deviation; IQR, Inter Quantile Range; CI, Confidence Interval. ^t^ Paired Samples *t*-test Statistic; ^z^ Wilcoxon Signed Ranks Test Statistic. * Statistically significant. Bold format indicates statistically significant results.

**Table 4:** Regional Tsk Comparison in Resting and Post-Activity Conditions.

		Rest (Mean ± SD/Median [IQR])	Post-Activity (Mean ± SD/Median [IQR])	Test Statistic	95% CI of Difference	*p*
Residual Limb	Patella	35.9 [33.8–36.4]	36.2 [34.2–36.3]	−0.73 ^z^	-	0.466
	Tibialis Anterior	35 ± 2	35 ± 2.4	−0.16 ^t^	[−0.86, 0.73]	0.876
	Distal	34.1 ± 2.4	34.5 ± 2.5	−1.12 ^t^	[−1.32, 0.39]	0.274
Intact Limb	Patella	34.8 [33.2–37]	34.6 [32.9–35.9]	−1.69 ^z^	-	0.092
	Tibialis Anterior	36.3 [35.9–36.5]	36.5 [36.2–36.6]	−2.03 ^z^	-	0.042 *
	Distal	33.7 ± 2.7	34.6 ± 2.3	−2.17 ^t^	[−1.75–0.04]	0.041 *

All measurements are expressed in degrees Celsius (°C). Abbreviations: SD, Standard Deviation; IQR, Inter Quartile Range; CI, Confidence Interval. ^t^ Paired Samples *t*-test Statistic; ^z^ Wilcoxon Signed Ranks Test Statistic. * Statistically significant. Bold format indicates statistically significant results.

## Data Availability

The original contributions presented in this study are included in the article. Further inquiries can be directed to the corresponding author.

## Abbreviations

The following abbreviations are used in this manuscript:

Tsk	Skin Temperature
TISEM	Thermographic Imaging in Sports and Exercise Medicine
SD	Standard Deviations
IQR	Interquartile Range
°C	Degrees Celsius
CI	Confidence Interval
